# Feeding controls H_2_S production in situ in high solid anaerobic digestion

**DOI:** 10.1186/s40643-022-00567-7

**Published:** 2022-08-04

**Authors:** Cen Ruxiang, Chen Ruiying, Pu Tianyun, Huang Chunyan, He Tengbing, Tian Guangliang

**Affiliations:** Resource Conservation and Germplasm Innovation in Mountainous Region (Ministry of Education), College of Agriculture, Institute of New Rural Development, Engineering Laboratory for Pollution Control and Resource Reuse Technology of Livestock and Poultry Breeding in Plateau Mountain (Guizhou Province), Guizhou University, Guiyang, 550025 China

**Keywords:** High solid, Anaerobic digestion, Short time-scale, H_2_S release, Volatile fatty acid

## Abstract

In this study, a high frequency monitoring method was used to assess how semi-continuous feeding affects H_2_S production in high solid anaerobic digestion. The results showed that H_2_S characteristics at a monitoring frequency of 1 point/3 h were different to that of 1 point/24 h, its concentration decreased from 3449 ± 227 mg/m^3^ at 0 h to 298 ± 45 mg/m^3^ at 3 h. H_2_S concentration was negatively correlated with volatile fatty acids (VFAs), and oxidation reduction potential (ORP). 72–82% of H_2_S reduction in the first 3 h resulted from the introduction of O_2_ during feeding, and 18–28% of that was closely related to the production of a large quantity of soluble acidic matter, such as VFAs. A more accurate H_2_S release model was established according to the content of VFAs. Totally, this study implies that feed carrying air is a promising method for in situ control of H_2_S production in anaerobic digestion.

**Graphical Abstract:**

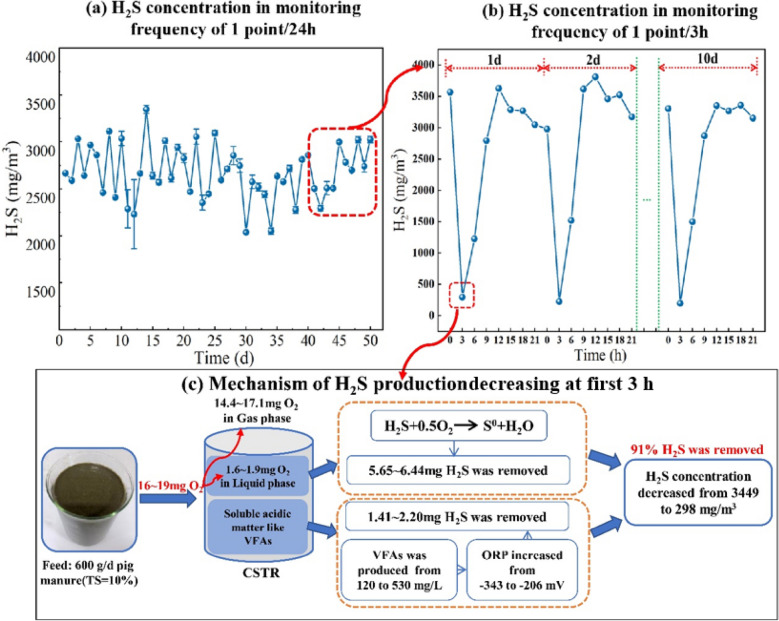

## Introduction

Annual livestock and animal manure production in China can reach 3.8 × 10^9^ t, with pig manure accounting for 47% of this total (~ 1.8 × 10^9^ t) (Liu et al. 2020). This large quantity of manure production results in serious environmental pollution. Anaerobic digestion (AD) can transform organic matter into energy and organic fertilizer, thereby achieving a reduction in waste and resource recovery that is of minimal environmental harm (Wafi et al. 2019; Adrover et al. 2020). Given these advantages, this technology has been widely used globally (Li et al. 2014; Hailu et al. 2020). AD is the main technology used within the treatment of animal manure (Tayibi et al. 2021; Wu et al. 2022). Research into anaerobic digestion of waste with a high solid content [total solids (TS) ≥ 10%] has become increasingly popular and further development of this technology in recent years has made it suitable for treating organic fertilizer wastes, such as animal manure, kitchen waste, and agricultural straw (Krishania et al. 2013; Ting et al. 2020; Mertins et al. 2022).

H_2_S gas is a product of AD and is poisonous, acidic, and malodorous. This gas also has a strong corrosive effect on pipelines, combustion chambers, and instruments (Oliveros-Muñoz et al. 2021). Therefore, measures must be taken to reduce the concentration of H_2_S gas in the biogas before using the biogas. Previous studies have proved that adding a small amount of O_2_ or air can effectively remove H_2_S in the anaerobic digestion system (Diaz et al. 2010; Lim et al. 2014; Yang et al. 2020). Diaz et al. used municipal sludge as raw material, and by supplying O_2_ to the anaerobic reactor, the removal rate of H_2_S in biogas can reach about 98%. In CSTR project operation, semi-continuous feed is used and the feed often brings in trace amounts of air; however, no study has been done on how semi-continuous feed affects the production of H_2_S.

H_2_S is mainly derived from the transformation of different forms of sulfur during AD, including sulfate reduction and decomposition of sulfur-containing protein (Yan et al. 2018). Some scholars have studied the release of H_2_S during AD. For example, Tian et al. (2020) showed that the average H_2_S concentration and production potential in a sequential batch AD test of food waste with 4.2% TS content under a monitoring frequency of 1 point /24 h were 1065 ± 267 ppm and 765 ± 163 g/t (TS), respectively. Yang and Deng (2020) found the H_2_S concentration to be 336 ± 150 ppm in semi-continuous AD of pig manure with a TS of 6% under a monitoring frequency of 1 point /24 h. Dai et al. (2017) showed that H_2_S concentration and production potential during the AD of activated sludge were 95 ± 13 ppm and 314.6 × 10^–4^ mL /g volatile solids (VS), respectively, under a monitoring frequency of 1 point /24 h. In general, these studies mainly focused on the emission characteristics of H_2_S under a low TS and long-time scale, such as a monitoring frequency of 24 h. In contrast, few studies have focused on the continuity of H_2_S production under a high solid content and AD over a short time scale.

Therefore, this study is the first to use a high frequency monitoring method to investigate how semi-continuous feeding affects H_2_S production in AD. The method of high frequency detection of 1 point/3 h was used to explore the relationship between the production characteristics of H_2_S and CH_4_ and physical and chemical factors in a high-solids Continuous Stirring Tank Reactor (CSTR) process.

## Materials and methods

### Materials

The pig manure used in the present study was obtained from the Chang Ping pig farm in Yuqing County, Guizhou Province, China. The inoculum was extracted from an AD test of pig manure in the laboratory. Table 1 shows the basic characteristics of pig manure and inoculum used in the present study.

**Table 1:** Basic characteristics of pig manure and inoculum used in the present study

Items	Swine manure	Inoculum
pH	7.09 ± 0.00	8.19 ± 0.00
Total solid content (%)	38.31 ± 0.25	9.43 ± 0.58
Volatile solid content (%)	79.85 ± 0.17	66.43 ± 0.32
Volatile fatty acid (mg/L)	2345 ± 184	–
Total sulfur (mg/L)	695 ± 25	–
S-sulfate (mg/L)	36.5 ± 0.4	–
S-total sulfide (mg/L)	252.9 ± 5.7	–
S-soluble sulfide (mg/L)	6.1 ± 0.7	–

– There is no test

### Experimental design

**Fig. 1:**
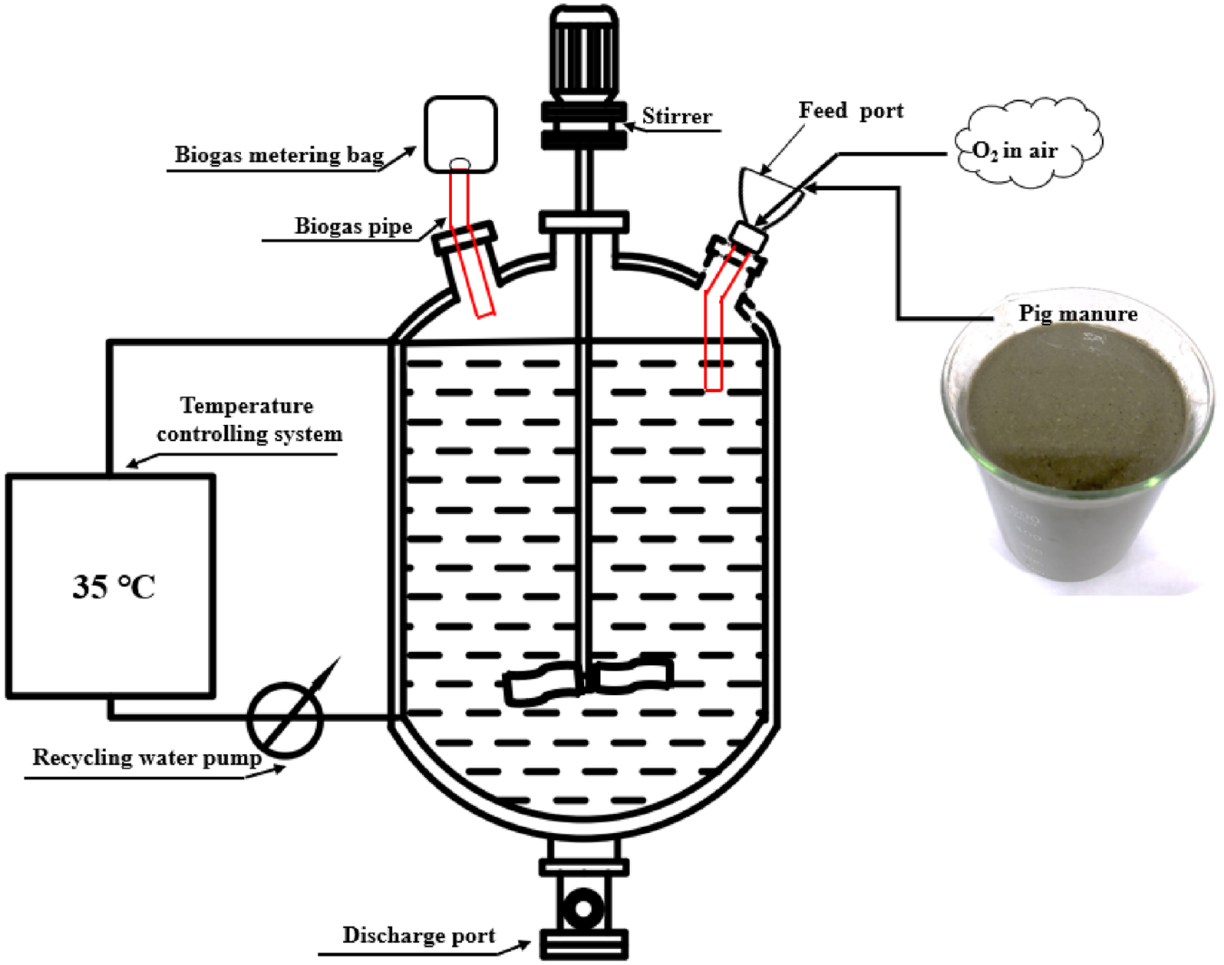
Schematic diagram of the CSTR system used in the present study

The CSTR reactor was cylindrical (Fig. 1) with a volume of 12 L, an effective volume of 9 L, and a Hydraulic Retention Time of 15 days. The CSTR was operated at a temperature of 35 ℃. Semi-continuous feeding was used, 600 mL pig manure (TS = 10%) was poured into CSTR in 3 min each day. Monitoring of H_2_S, CH_4_, and volatile fatty acids was initiated at the start of the reactor. Samples of gas and fermentation broth were collected daily at 0 h (before feeding) and at 3, 6, 9, 12, 15, 18, and 21 h (after feeding). Biogas collected using a tin foil air bag was used for the determination of CH_4_, H_2_S, and O_2_. The samples were evenly transferred into a 50 mL sterile tube and stored at − 25 ℃ for the determination of physical and chemical properties.

### Analytical methods

The concentration of H_2_S in biogas was determined by gas chromatography (GC 1120, Shanghai Hengping). Injection method: 5 mL of biogas sample containing H_2_S was injected into the injection port with a syringe, and the injection volume was controlled to 0.5 mL by the quantification ring in the chromatograph. Operating conditions of the chromatograph: the column was a capillary column (SH-Rtx-1,60 m × 0.53 mm × 0.5um), the detector was a Flame Photometric Detector (FPD), the temperatures of the column, the inlet and the detector were 60, 200 and 250 °C, respectively. Helium was used as the carrier gas. Calculation method: The concentration of H_2_S in biogas is calculated according to area external standard method.

The daily output of biogas was measured using an LMP-1 wet type anticorrosive gas flow meter (Chongqing Jieheng Peristaltic Pump Co., Ltd.). Measurements of CH_4_ and O_2_ were conducted using a gas chromatograph under the thermal conductivity detector (TCD) method. TS and VS were determined using the gravimetric method (Choudhury and Lansing 2020). pH, oxidation reduction potential (ORP), and dissolved oxygen (DO) were measured using a Hach water quality monitor. Ammonium nitrogen (NH_4_^+^–N) was determined using the Nessler's reagent colorimetric method. VFAs were determined by gas chromatography [gas chromatograph + flame-ionization detection (FID) + DWAX capillary column]. Sulfate and total sulfur content were measured by barium chromate spectrophotometry, methylene blue colorimetry, and an elemental analyzer, respectively. The S-total sulfides was determined by methylene blue spectrophotometric method with original sludge, while S-soluble sulfide was determined by methylene blue spectrophotometric method with liquid produced by filtration of original sludge through 0.45 um microporous filter membrane (Tian et al. 2020).

### H_2_S emission formula based on the sulfide equilibrium

Equation (1) shows the sulfide ionization equilibrium model and its derivation process (Tian et al. 2020):

1 $${\mathrm{C}}_{{\mathrm{H}}_{2}\mathrm{S}}=34{\mathrm{S}}_{\mathrm{T}}/\left(32\left(1+\frac{{\mathrm{K}}_{\mathrm{S}1}}{{10}^{-\mathrm{pH}}}+\frac{{\mathrm{K}}_{\mathrm{S}1}{\mathrm{K}}_{\mathrm{S}2}}{{10}^{-2\mathrm{pH}}}\right)\right)$$

In Eq. (1), C_H2S_ is the concentration of H_2_S in the liquid phase (mg/m^3^), Ks_1_ and Ks_2_ are the second and first equilibrium constants of sulfide, with values of 7.1 × 10^–15^ and 1.3 × 10^–7^, respectively, and S_T_ represents soluble sulfide (mg/m^3^) in fermentation broth.

Equation (2) shows the formula for the prediction of H_2_S concentration in the gas phase:

2 $$\mathrm{C}={\mathrm{EC}}_{{\mathrm{H}}_{2}\mathrm{S}}$$

In Eq. (2), C is the concentration of H_2_S in the gas phase (mg/m^3^) and E is the Henry coefficient (0.686 kPa at 35 ℃).

## Results and discussion

### Characteristics of Biogas, CH_4_, H_2_S and O_2_

#### Biogas and CH_4_

**Fig. 2:**
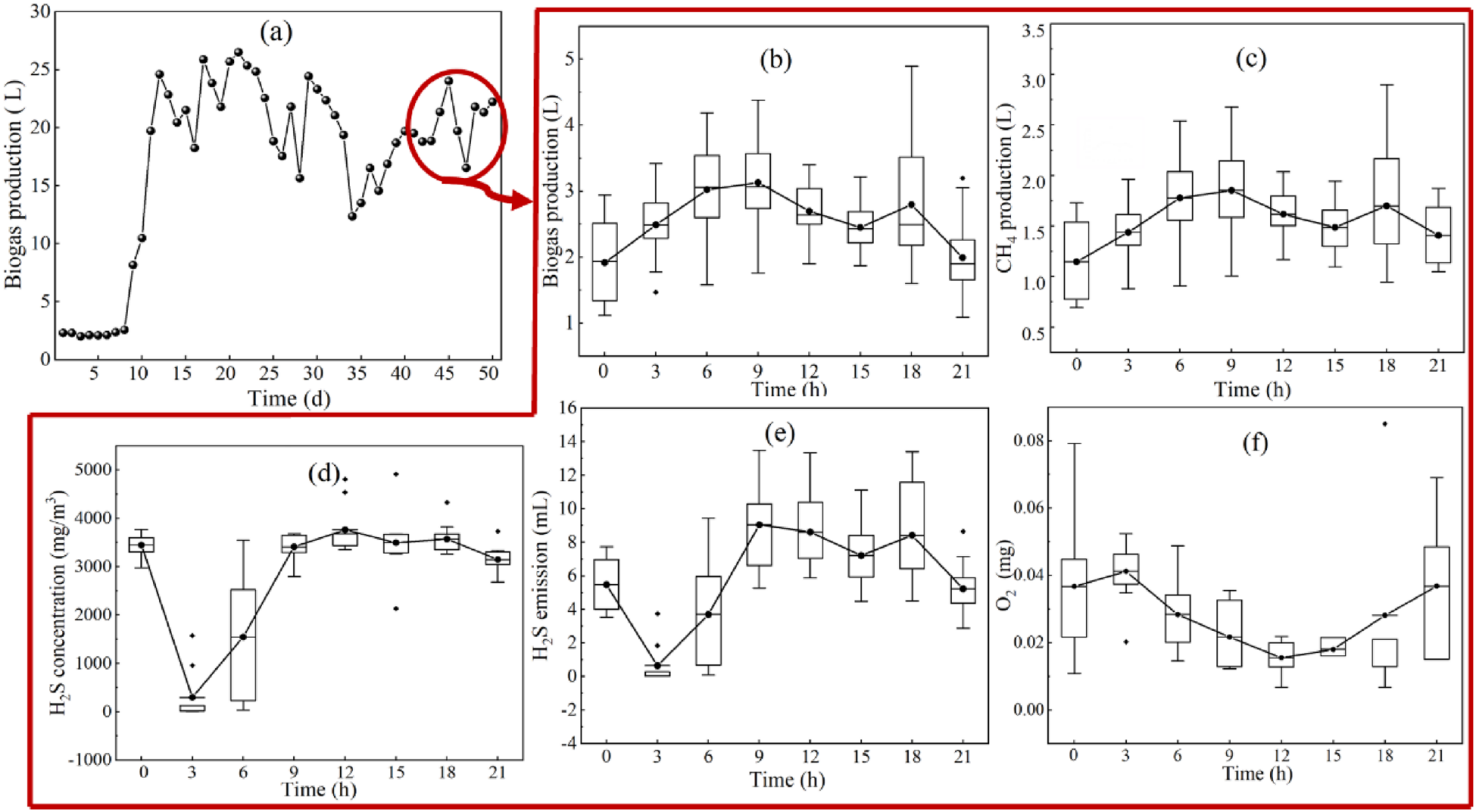
Characteristics of fermentation gas generated by a CSTR: **a** daily production of 50 d biogas; **b** production of biogas under a monitoring frequency of 1 point/3 h; **c** CH_4_ production; **d** H_2_S concentration; **e** H_2_S production; **f** O_2_ mass in biogas. Note:0 h means the time before feeding and the time at 24 h

As shown in Fig. 2a, there was an increasing trend in biogas production during AD over 50 days from day 1 to day 10 with a yield of 2.02–10.48 L/d. Biogas production then stabilized from the day 11 to day 50 at a high average yield of 20.62 ± 3.43 L/d. During this stage, the average CH_4_ and CO_2_ contents of biogas were 63.21 ± 0.16% (v/v) and 36.56 ± 0.06% (v/v), respectively. Biogas and H_2_S were monitored at 1 point /3 h after 40 days of fermentation. As shown in Fig. 2b, c, biogas and CH_4_ showed upward trends during the initial stage of feeding at rates of 1.92 ± 0.61 L/3 h and 1.15 ± 0.36 L/3 h, respectively, at 0 h (before feeding) to 3.13 ± 0.13 L/3 h and 1.85 ± 0.45 L/3 h, respectively, at 9 h (After feeding), after which the gas production rate showed a gradual downward trend. The rates of biogas and CH_4_ production were 1.99 ± 0.67 L/3 h and 1.41 ± 0.31 L/3 h, respectively, by 21 h. The average rates of biogas and CH_4_ production were 2.56 ± 0.41 L/3 h and 1.55 ± 0.21 L/3 h, respectively.

#### H_2_S and O_2_

As shown in Fig. 6a, the average concentration and release potential of H_2_S were 3,335 ± 352 mg/m^3^ and 1,265 ± 578 g/t (TS), respectively, at a monitoring frequency of 1 point/24 h. There were clear differences in the changes in H_2_S concentration under a monitoring frequency of 1 point/3 h to that under a monitoring frequency of 1 point/24 h. As shown in Fig. 2d, the concentration of H_2_S decreased rapidly at the beginning of feeding, from 3449 ± 227 mg/m^3^ at 0 h (before feeding) to 298 ± 45 mg/m^3^ at 3 h (after feeding), following which it gradually increased and stabilized at 9 h at a concentration of 3149 ± 277 mg/m^3^–3763 ± 472 mg/m^3^ from 9 to 21 h. A similar trend was noted in the production of H_2_S (Fig. 2e). The results of the present study were consistent with that of Huang et al. (2014) who show showed that the concentration of H_2_S in the methanogenic phase of two-phase AD of food waste first increases and then decreases after feeding, with a maximum H_2_S concentrations of 437 mg/m^3^ and 175 mg/m^3^ between 8–9 h, and 23–24 h, respectively. The low concentration of H_2_S in the study by Huang et al. (2014) may be due to the acidified phase in the two-phase anaerobic reactor leading to the early release of a portion of the H_2_S.

As shown in Fig. 2f, O_2_ content in biogas components gradually decreased as AD progressed, with a concentration of 1.44% at 3 h, decreasing to 0.99% at 6 h, further decreasing to 0.76% at 9 h, and stabilizing at 0.49–0.69% at 12–21 h. According to these results, the content of O_2_ in biogas increased by 15–17 mg, whereas daily atmospheric O_2_ carried by the feed was 16–19 mg. Therefore, the increased O_2_ in the biogas originated from atmospheric O_2_ carried by the feed.

### Characteristics of pH, ORP, DO and VFAs

**Fig. 3:**
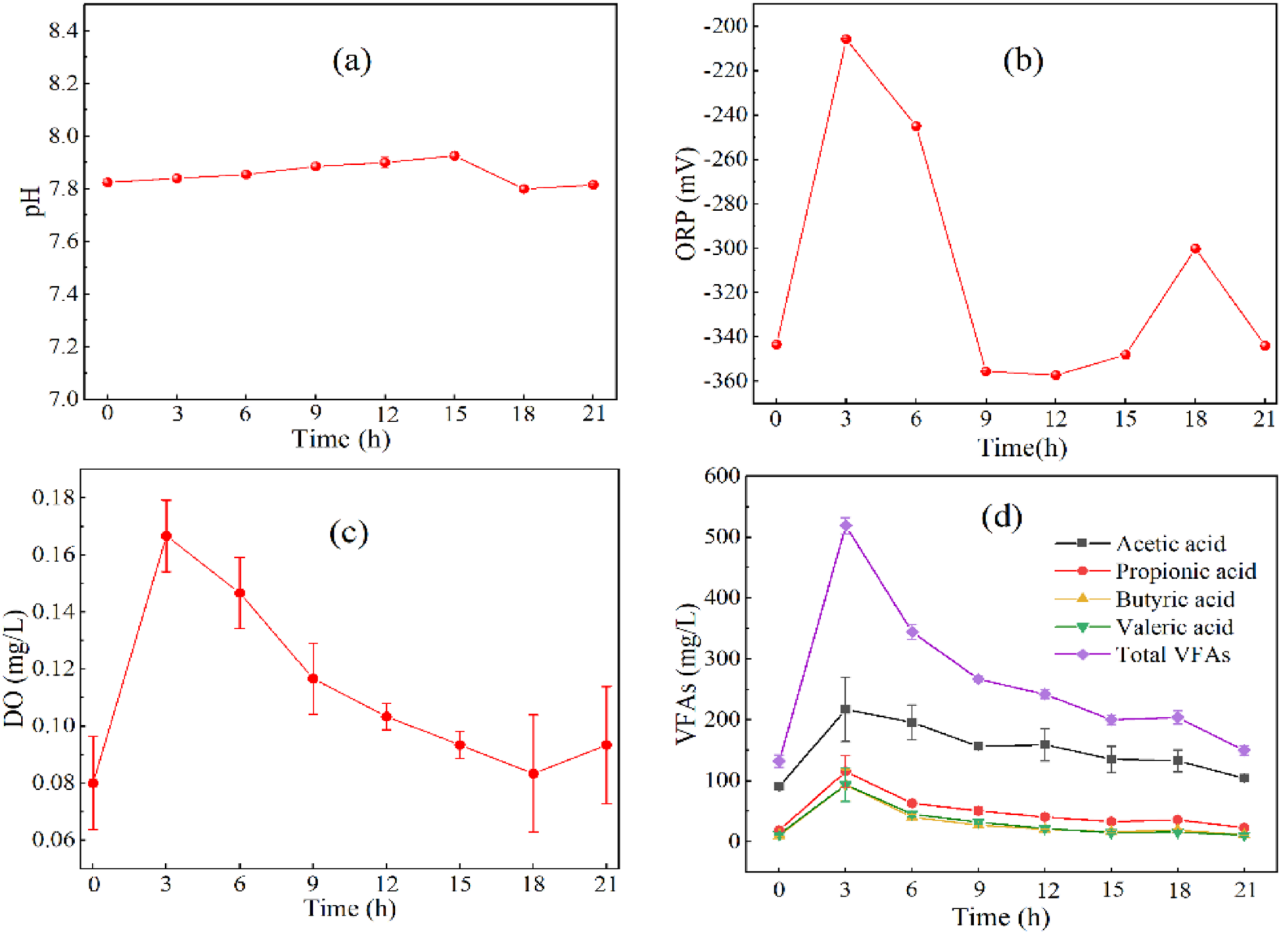
Characteristics of pH, ORP, DO, and VFAs within the anaerobic fermentation of pig manure with a high solid. **a** pH; **b** ORP; **c** DO; **d** total VFAs

As shown in Fig. 3a, a stable pH of the CSTR system with high solid pig manure was observed, ranging from 7.80 to 7.93, remaining in a range suitable for the growth of methanogens and indicating a healthy state of fermentation (Chotinath Vongvichiankul 2017). As shown in Fig. 3b, there was a rapid increase in ORP in the reactor within 3 h after feeding, rising from − 343.4 ± 0.8 mV at 0 h to − 205.8 ± 0.5 mV at 3 h. ORP gradually decreased with the progression of AD from − 244.9 ± 0.1 mV at 6 h to − 355.5 ± 0.2 mV at 9 h, following which it stabilized at − 357.3 ± 0.2 mV to − 300.1 ± 0.1 mV. As shown in Fig. 3c, there was a rapid increase in DO in the reactor within 3 h after feeding, rising from 0.08 ± 0.02 mg/L at 0 h to 0.17 ± 0.01 mg/L at 3 h. DO tended to stabilize with progression of AD, maintaining a concentration of between 0.08 ± 0.02 mg/L–0.12 ± 0.01 mg/L, indicating a gradual absorption of O_2_ by the fermentation broth.

As shown in Fig. 3d, four VFAs, namely, acetic acid, propionic acid, butyric acid, and valeric acid, were produced in large quantities during the initial 3 h of AD. Acetic acid concentration increased from 90 ± 1 mg/L at 0 h to 217 ± 52 mg/L, propionic acid increased from 19 ± 1 mg/L to 115 ± 26 mg/L, butyric acid increased from 11 ± 2 mg/L to 93 ± 25 mg/L, and valeric acid increased from 12 ± 1 mg/L to 93 ± 28 mg/L. Acetic acid was the main component of total VFAs (42% of total VFAs). The concentrations of all VFAs gradually decreased with progression of AD. In a study like to the current study, Andreides et al. (2021) used a solution of ricotta cheese as material for sequential batch AD, with the results of their study showing that VFAs were almost completely consumed after 8 h. Within the present study, the concentration of VFAs of the feed sample at the start of AD was relatively high at 2345 ± 184 mg/L. On the other hand, a large quantity of dissolved organic matter contained in raw materials can be rapidly transformed into VFAs by microorganisms, resulting in an increase in the initial concentration of VFAs of fermentation (Yin et al. 2021). Since a large number of VFAs were transformed into CH_4_ and CO_2_ as fermentation progressed, the concentration of VFAs gradually decreased, consistent with the results of previous studies on the AD of pig and chicken manure (Ao et al. 2021; Huang et al. 2016).

There was negligible change in the concentration of NH_4_^+^–N in the present study, remaining between 1766 and 2000 mg/L.

### The relationship between ORP, DO, VFAs and H_2_S

**Fig. 4:**
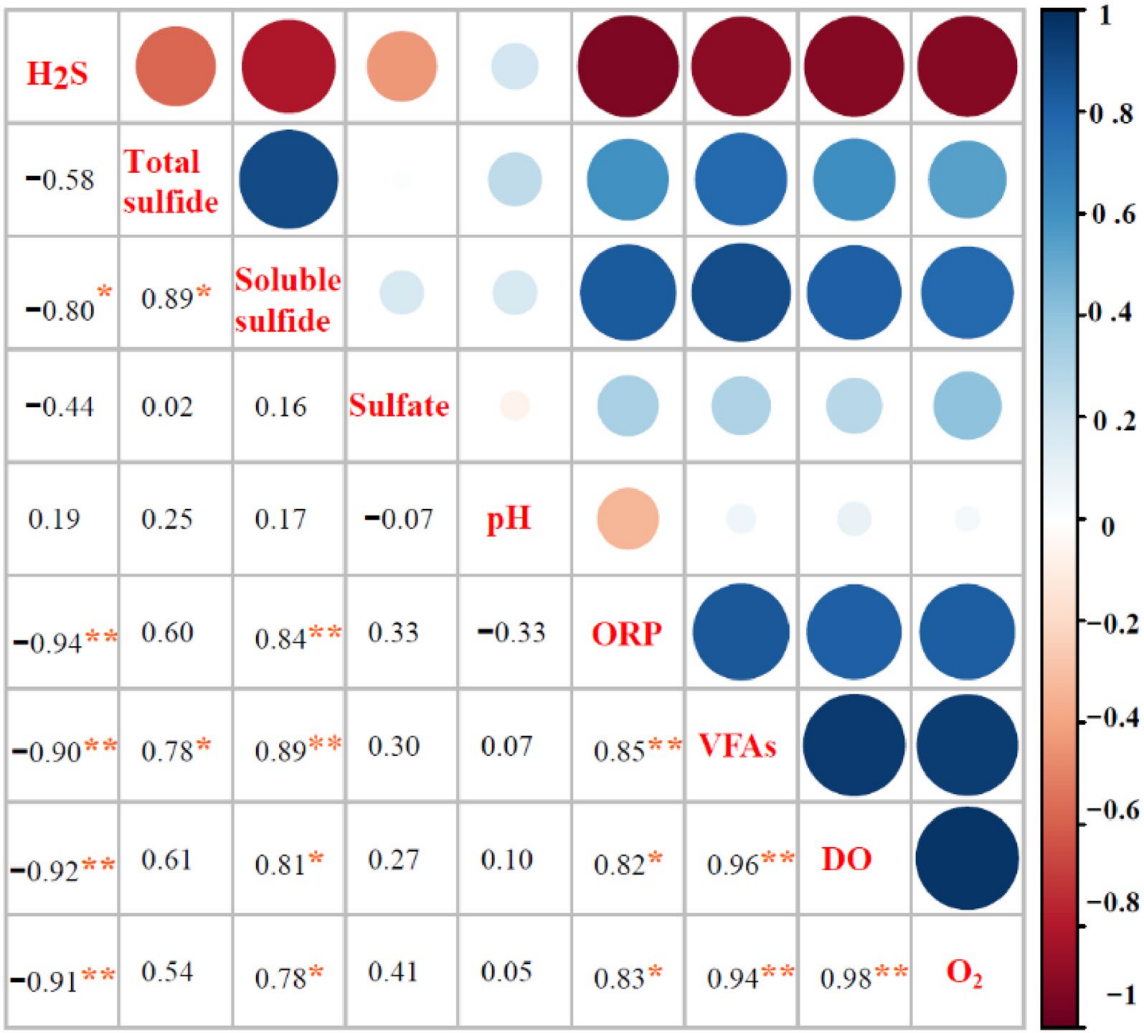
Pearson correlation heatmap between physiochemical characteristics and H_2_S concentration. (Note Significance level: * *P* < 0.05, ** *P* < 0.01, n = 8)

The present study further examined the correlation between physical and chemical properties of fermentation broth and H_2_S concentration using Pearson correlation analysis. As shown in Fig. 4, the concentration of H_2_S was negatively correlated with O_2_ content, ORP, DO and VFAs in biogas (*P* < 0.01), whereas ORP was positively correlated with VFAs (*P* < 0.01). The results of the present study showed that an increase in ORP in broth during the fermentation of pig manure from − 357 mV to − 205 mV resulted in a decrease in H_2_S concentration in biogas from 3449 mg/m^3^ to 298 mg/m^3^. Consistent with the results of the present study, Nghiem et al. (2014) demonstrated a decrease in H_2_S concentration in biogas from 6,000 ppm to < 30 ppm with an increase in ORP from − 320 mV to − 270 mV during AD of sewage. Since the pH of the reactor was maintained within a small range of between 7.80 and 7.93 in the present study, it can be asserted that pH had no significant influence on H_2_S concentration (*R* = 0.19). Past studies have shown that the initial pH of sludge has an impact on H_2_S generation during AD of abattoir wastewater. For example, Yan et al. (2018) showed that an initial increase in pH of sludge from 6.5 to 8.0 resulted in an increase in biogas production of 10.1%, whereas H_2_S concentration decreased by 44.7%.

### Variations in different forms of sulfur

**Fig.5:**
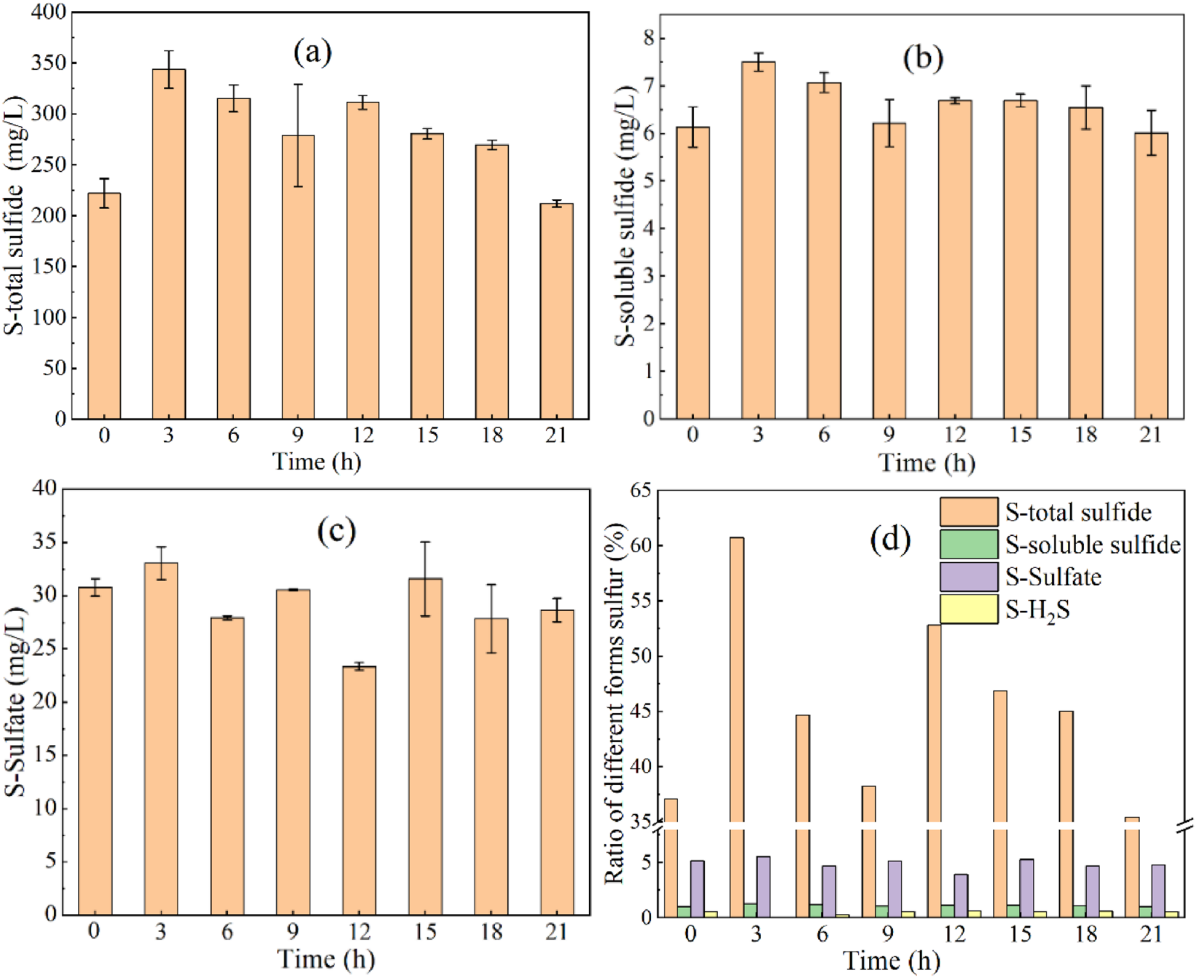
Changes in the forms of sulfur before and after feeding: **a** S-total sulfide; **b** S-soluble sulfide; **c** S-sulfate; **d** percentage value in the figure is the proportion of each form of sulfur to total sulfur

As shown in Table 1, there was an average concentration of total sulfur in raw pig manure of 695 ± 93 mg/L, whereas total sulfur in fermentation liquid was 629 ± 43 mg/L, indicating that fermentation did not lead to significant loss in sulfur. As shown in Fig. 5a, S-total sulfide in the reactor increased from 222 ± 14 mg/L at 0 h to 343 ± 18 mg/L at 3 h, following which it gradually decreased. The concentration of S-total sulfide at 21 h was 212 ± 3 mg/L. As shown in Fig. 5d, Stotal sulfide accounted for 36% to 60% of total sulfur. Therefore, S-total sulfide constituted the main chemical form of sulfur.

As shown in Fig. 5b, there was a rapid increase in S-soluble sulfide in the reactor, from 6.1 ± 0.7 mg/L to 7.5 ± 0.2 mg/L at 3 h before fermentation, following which it gradually decreased from 7.1 ± 0.2 mg/L at 6 h to 6.0 ± 0.5 mg/L at 21 h. As shown in Fig. 5d, S-soluble sulfide accounted for 0.9–1.3% of total sulfur. A significant negative correlation was noted between S-soluble sulfide and H_2_S concentration (*P* < 0.05), inconsistent with the sulfide ionization equilibrium model. This result could be attributed to oxidation of H_2_S in the liquid phase during the transfer to the gas phase.

As shown in Fig. 5c, there was a negligible difference in S-sulfate concentration at 0 h at an average concentration of 29.2 ± 2.8 mg/L. Table 1 shows that the S-sulfate concentration of raw material was 36.4 ± 0.3 mg/L, indicating no obvious reduction of sulfate in the fermentation system. The proportion of S-sulfate in total sulfur was 3.9–5.5%, whereas the proportion of H_2_S in total sulfur was 0.0–0.6%.

### The mechanism of H_2_S emission

#### Sulfide ionization equilibrium model

**Fig. 6:**
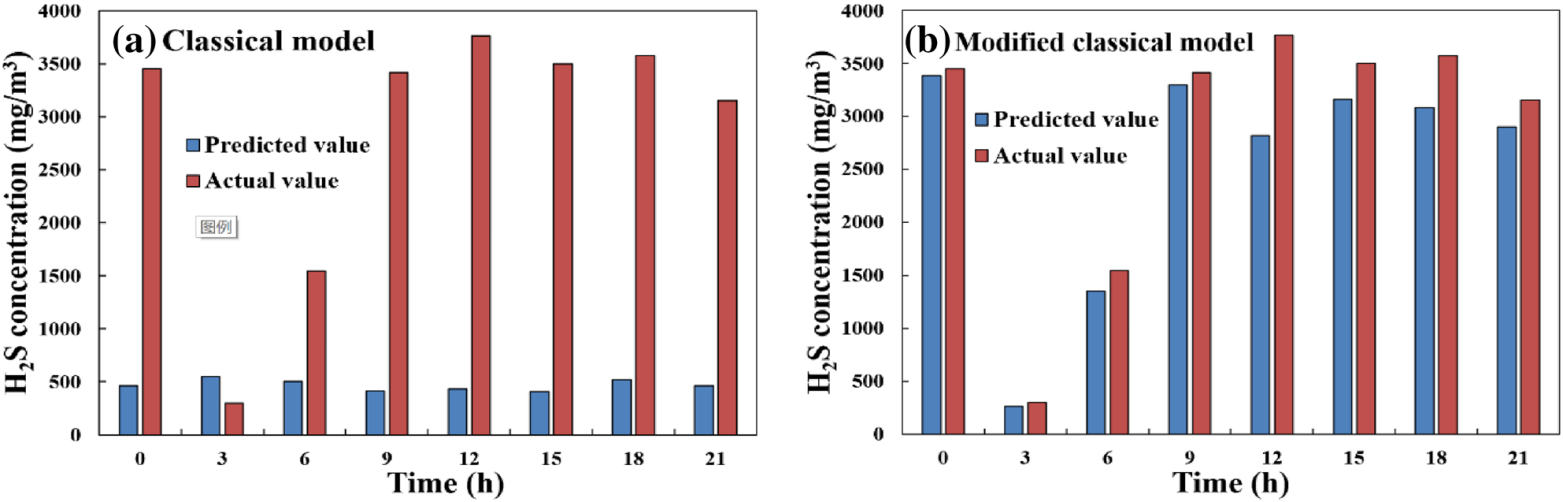
Predicted and measured values of H_2_S in AD of pig manure with high total solids

The concentration of H_2_S in the fermentation broth was calculated by applying the concentration of soluble sulfide (Fig. 5b) and pH (Fig. 3a) to the sulfide ionization equilibrium model (Eq, 1). Then H_2_S concentration in the gas phase was then calculated according to Henry's coefficient at 35 ℃ using Eq. 2. As shown in Fig. 6a, besides for at 3 h, the predicted value was only 12–31% of the observed value, indicating an inaccurate prediction.

As shown in Fig. 4, there was very significant negative correlation between the concentration of H_2_S and total VFAs (R =  − 0.9, *P* < 0.01). Therefore, the present study attempted to modify the sulfide ionization equilibrium model using the concentration of total VFAs. Under a conversion factor of 5.5, the modified model was: $\mathrm{C}={5.5\mathrm{B}}_{VFA}{\mathrm{EC}}_{{\mathrm{H}}_{2}\mathrm{S}}$(note: B_VFA_ represents the concentration of total VFAs). As shown in Fig. 6b, the accuracy of predictions improved to 72–99%. In summary, the concentration of total VFAs indicated that the correction coefficient of the classical sulfide ionization equilibrium model is 5.5, and application of this coefficient significantly improved the accuracy of the model.

#### Mechanism of H_2_S release

**Fig. 7:**
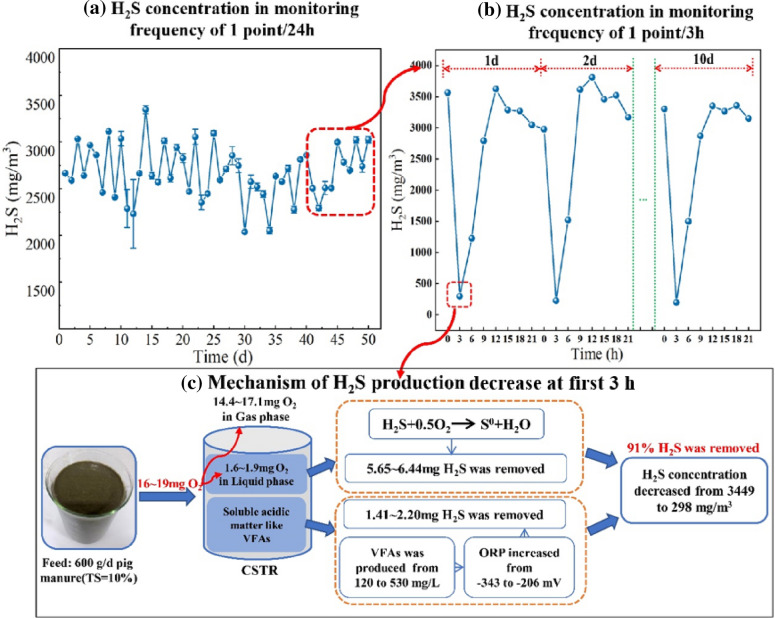
Proposed characteristics and mechanism of H_2_S emission at a 3 h time scale according to the results of the present study

As shown in Fig. 7a, b, the changes in H_2_S concentration under a monitoring frequency of 1 point/3 h were significantly different from those under a monitoring frequency of 1 point/24 h. This result can be ascribed to the rapid decrease in H_2_S 3 h after feeding, which can be attributed to two processes, described below.

The first process is the direct oxidation of H_2_S by O_2_. As shown in Figs. 2f and 4, and the previous analysis, 70–80 mg of air was assimilated by the fermenter during each feed, of which 16–19 mg was O_2_. Approximately 90% of the O_2_ was transferred to the gas phase, with the remainder transferred to the fermentation broth (1.6–1.9 mg), with this portion of O_2_ available for conversion of H_2_S into zero-valent sulfur (Diaz et al. 2010; Mahdy et al. 2020). This resulted in the removal of 5.49–6.28 mg of H_2_S produced in the first 3 h, accounting for 72% to 82% of the total removal (7.85 mg) (Fig. 7c).

The second process is the increase in ORP resulting from the increase in VFAs and other acidic organic matter during the early stage of fermentation. This led to the oxidation of sulfide. As shown in Fig. 4 and the previous analysis, VFAs had a very significant negative correlation with H_2_S concentration (*R* = 0.9, *P* < 0.01) and a very significant positive correlation with ORP (*R* = 0.85, *P* < 0.01). The concentration of VFAs increased rapidly (from 132 ± 10 mg/L to 519 ± 13 mg/L) within 3 h of feeding. This result could mainly be attributed to the higher concentration of VFAs in the feed samples of 2,345 ± 184 mg/L, followed by dissolution of VFAs in the raw materials of fermentation as organic matter was rapidly converted into VFAs during the initial stage. On the other hand, the negligible change in DO concentration in the fermentation broth (Fig. 3c) of between 0.08 ± 0.02 mg/L and 0.12 ± 0.01 mg/L was insufficient to affect ORP. Based on the above analysis, the present study proposed that the increase in volatile acids and other acidic organic substances was the main driver of the increase in ORP, which in turn led to the decrease in H_2_S. Like the present study, Nghiem et al. (2014) showed that H_2_S concentration in biogas decreased from 6,000 ppm to 30 ppm as ORP in sludge increased from − 320 mV to − 270 mV. In summary, an additional reason for the rapid decline in H_2_S concentration during the first 3 h was the increase in ORP due to the presence of a large quantity of acidic organic matter in the early stage, such as VFAs.

## Conclusions

The present study that variation in H_2_S in a CSTR with high solid pig manure under a monitoring frequency of 1 point /3 h was clearly different from that under a monitoring frequency of 1 point /24 h. Specifically, the concentration of H_2_S rapidly decreased within the first 3 h of fermentation, from 3449 ± 227 mg/m^3^ at 0 h (before feeding) to 298 ± 45 mg/m^3^ at 3 h (after feeding), following which it rapidly increased between 4 and 8 h, and stabilized to between, 3149 ± 277 mg/m^3^ and 3763 ± 472 mg/m^3^ from 9 to 21 h. O_2_ contents in biogas, VFAs, and ORP were negatively correlated with H_2_S concentration (*P* < 0.01). Mass balance analysis showed that the decrease in H_2_S in the first 3 h could be partly attributed to oxidization of H_2_S by O_2_ carried by the feed, and partly to the increase in ORP due to the increase in acidic organic matter, such as VFAs, leading to the oxidization of H_2_S. The accuracy of sulfide ionization equilibrium model was improved by considering the concentration of VFAs. The results of the present study can act as a reference for further research into the regulation of H_2_S in situ in high solid AD by controlling the feed carrying O_2_.

## Data Availability

Not applicable.
